# Identifying Mild Cognitive Impairment in Parkinson’s Disease With Electroencephalogram Functional Connectivity

**DOI:** 10.3389/fnagi.2021.701499

**Published:** 2021-07-01

**Authors:** Min Cai, Ge Dang, Xiaolin Su, Lin Zhu, Xue Shi, Sixuan Che, Xiaoyong Lan, Xiaoguang Luo, Yi Guo

**Affiliations:** ^1^Department of Neurology, Shenzhen People’s Hospital, The Second Clinical Medical College, Jinan University, Shenzhen, China; ^2^The First Affiliated Hospital, Southern University of Science and Technology, Shenzhen, China; ^3^Department of Medical, The Fourth People’s Hospital of Chengdu, Chengdu, China; ^4^MOE Key Lab for Neuroinformation, Chengdu Mental Health Center, The Clinical Hospital of Chengdu Brain Science Institute, University of Electronic Science and Technology of China, Chengdu, China; ^5^Shenzhen Bay Laboratory, Gladstone Institute of Neurological Disease, Shenzhen, Guangdong, China

**Keywords:** Parkinson’s disease, cognitive impairment, electroencephalography, functional connectivity, theta frequency band

## Abstract

**Objective:**

Cognitive impairment occurs frequently in Parkinson’s disease (PD) and negatively impacts the patient’s quality of life. However, its pathophysiological mechanism remains unclear, hindering the development of new therapies. Changes in brain connectivity are related to cognitive impairment in patients with PD, with the dorsolateral prefrontal cortex (DLPFC) being considered the essential region related to PD cognitive impairment. Nevertheless, few studies have focused on the global connectivity responsible for communication with the DLPFC node, the posterior division of the middle frontal gyrus (PMFG) in patients with PD; this was the focus of this study.

**Methods:**

We applied resting-state electroencephalography (EEG) and calculated a reliable functional connectivity measurement, the debiased weighted phase lag index (dWPLI), to examine inter-regional functional connectivity in 68 patients with PD who were classified into two groups according to their cognitive condition.

**Results:**

We observed that altered left and right PMFG-based functional connectivity associated with cognitive impairment in patients with PD in the theta frequency bands under the eyes closed condition (*r* = −0.426, *p* < 0.001 and *r* = −0.437, *p* < 0.001, respectively). Exploratory results based on the MoCA subdomains indicated that poorer visuospatial function was associated with higher right PMFG-based functional connectivity (*r* = −0.335, *p* = 0.005), and poorer attention function was associated with higher left and right PMFG-based functional connectivity (*r* = −0.380, *p* = 0.001 and *r* = −0.256, *p* = 0.035, respectively). Further analysis using logistic regression and receiver operating characteristic (ROC) curves found that this abnormal functional connectivity was an independent risk factor for cognitive impairment [odds ratio (OR): 2.949, 95% confidence interval (CI): 1.294–6.725, *p* = 0.01 for left PMFG; OR: 11.278, 95% CI: 2.578–49.335, *p* = 0.001 for right PMFG, per 0.1 U], and provided moderate classification power to discriminate between cognitive abilities in patients with PD [area under the ROC curve (AUC) = 0.770 for left PMFG; AUC = 0.809 for right PMFG].

**Conclusion:**

These preliminary findings indicate that abnormal PMFG-based functional connectivity patterns associated with cognitive impairment in the theta frequency bands under the eyes closed condition and altered functional connectivity patterns have the potential to act as reliable biomarkers for identifying cognitive impairment in patients with PD.

## Introduction

Parkinson’s disease (PD) is clinically characterized by the presence of motor symptoms, including bradykinesia, tremor, rigidity, and postural instability (Sveinbjornsdottir, 2016). However, non-motor symptoms are currently receiving increased attention as they often precede the development of motor symptoms and can impose a significant burden on the patient, affecting independence in performing daily activities and negatively impacting the quality of life, independent of any motor symptoms (Tolosa et al., 2009). In particular, cognitive impairments frequently occur in patients with PD. Longitudinal studies have found that 10% of patients display cognitive deficits within three years of diagnosis; these numbers rise to 46% within 10 years and can reach as high as 83% within 20 years (Hanagasi et al., 2017). Therefore, it is essential to explore objective and non-invasive predictive biomarkers for this condition in its early stages.

The pathophysiological mechanisms responsible for cognitive impairment in patients with PD remain unclear. With the development of functional network science and neuroimaging approaches, it is now recognized that the pathophysiological disruptions associated with the development of neuropsychiatric disorders are rarely confined to a single region of the brain; rather, it is now known that there are alterations in functional connectivity within brain networks (Kehagia et al., 2013). Moreover, a dual syndrome hypothesis has been widely accepted, which distinguishes early frontal, dopaminergic-dependent dysexecutive syndrome, and later dopamine-independent posterior cortical syndrome (Fornito et al., 2015). Compared with other functional imaging methods, electroencephalography (EEG) has several advantages, predominantly its high temporal resolution, where specific frequency analysis enables precise investigation of connectivity changes (Donner and Siegel, 2011). Several EEG studies have found brain connectivity changes associated with cognitive impairment in patients with PD (Bertrand et al., 2016; Hassan et al., 2017; Chaturvedi et al., 2019; Sanchez-Dinorin et al., 2021), which might present potential markers for cognitive dysfunction, although various alterations in different frequency bands were observed in these studies. However, volume conduction issue resulting from the transient propagation of the electric fields engendered by the primary current source to most of the scalp sensors may influence connectivity estimates of EEG. Due to this linear mixing of signals from different brain regions detected by the same sensor, common methods used for functional connectivity evaluation (coherence or mutual information) may lead to the identification of transparent functional connections that do not accurately reflect the interactions between brain regions (Vinck et al., 2011). Several new functional connectivity assessment techniques have been developed to minimize the effects caused by volume conduction. In particular, the weighted phase lag index (wPLI), which weighs the contribution of the observed phase lead or lags by the magnitude of the imaginary component of the cross-spectrum, is less sensitive to additive volume-conducted noise sources (Lau et al., 2012). However, this measure has a positive bias. To solve this problem, Vinck et al. proposed a debiased estimator of the squared WPLI [i.e., dWPLI (Vinck et al., 2011)] that has been frequently applied to assess EEG data (Hardmeier et al., 2014; Wang et al., 2017).

Although there are many possible pathophysiological explanations for the correlation between EEG changes and the cognitive state of PD patients, the exact impaired circuitry responsible for these alterations is not clear. The dorsolateral prefrontal cortex (DLPFC), which transmits afferent projections to the caudate and putamen and is involved in higher-order cognitive functions, has been considered the essential region related to cognitive impairments in PD (Polito et al., 2012; Gratwicke et al., 2015; Rosero Pahi et al., 2020). Ko et al. demonstrated that stimulation of the right DLPFC, which is the most “sensitive” area related to PD-cognitive deficit-related metabolic pattern (PDCP), may normalize the altered PDCP network (Ko et al., 2014). In addition, non-invasive brain stimulation (NIBS) of the left DLPFC has been found to improve cognitive function in patients with PD (Beheshti and Ko, 2021). The posterior division of the middle frontal gyri (PMFG, left and right) are key nodes in the DLPFC that are widely used as accessible cortical stimulation sites for NIBS (Chen et al., 2013). In terms of the concept of brain networks, abnormal activity of a certain circuit component may modify its communication with other network components, which could be interpreted as modulation of functional connectivity throughout the brain (Gratwicke et al., 2015). Correspondingly, investigating the functional connectivity interacting with a focal targeted brain region may facilitate the development of a mechanism-specific intervention by identifying appropriate subgroups of clinical trials and providing objective measures of disease inhibition. Few studies have focused on global connectivity communicating with the particular region related to cognitive impairment in PD patients using resting-state EEG. Therefore, we hypothesized that the functional connectivity pattern of the PMFG might be a potential marker for identifying cognitive dysfunction in patients with PD.

To test this hypothesis, we applied resting-state EEG to measure potential PMFG-based functional connectivity (dWPLI) differences between PD patients with mild cognitive impairment (PD-MCI) and PD patients with normal cognition (PD-NC). In particular, we investigated whether abnormal functional connectivity was associated with cognitive function, and furthermore, whether this could discriminate between PD-MCI and PD-NC patients.

## Materials and Methods

### Subjects

This study was approved by the Institutional Research Ethics Committee of Shenzhen People’s Hospital and adheres to the recommendations of the Declaration of Helsinki. All PD patients were recruited from the inpatient or outpatient clinic at Shenzhen People’s Hospital from March 2019 to November 2020, and written informed consent was obtained from each participant. The inclusion criteria were as follows: (1) age of 40–80 years; (2) idiopathic PD based on the UK Parkinson’s Disease Society Brain Bank criteria (Hughes et al., 1992); (3) self-reported right-handedness. The exclusion criteria included the following: (1) documentation of serious organ dysfunction; (2) history of psychiatric disorders such as major depression, generalized anxiety disorder, or schizophrenia; (3) severe PD [Hoehn&Yahr (H&Y) scale stage > 4]; (4) any kind of dementia; (5) changes in antiparkinsonian medication within one month prior to enrolment and use of drugs that may influence EEG.

Each patient underwent a series of clinical evaluations that assessed both motor and non-motor symptoms. Motor disability associated with PD was evaluated with the Unified Parkinson’s Disease Rating Scale-III (UPDRS-III) (Movement Disorder Society Task Force on Rating Scales for Parkinson’s Disease., 2003), and disease severity was assessed according to the H&Y stage (Hoehn and Yahr, 1967). The Hamilton Anxiety Rating Scale (HAMA) (Hamilton, 1959) and the Hamilton Depression Rating Scale (HAMD) (Hamilton, 1967) were used to assess anxiety and depression states, respectively. Sleep quality was estimated using the Pittsburgh Sleep Quality Index (PSQI) (Buysse et al., 1989).

Gobal cognitive abilities were evaluated using the Montreal Cognitive Assessment (MoCA) (Nasreddine et al., 2005). According to level I of the PD-MCI diagnostic criteria proposed by the Movement Disorder Society (Litvan et al., 2012), PD patients with a MoCA score of 24 or above (Lucza et al., 2015) and normal functionality were identified as PD-NC. Patients with a MoCA score below 24 and preserved functionality were identified as PD-MCI. All neurological functional assessments were conducted in the “on” status, and the individual drugs used were converted to the levodopa equivalent daily dose (LEDD) (Tomlinson et al., 2010).

### EEG Acquisition and Preprocessing

Eight-minute resting-state EEGs of both the eyes closed (EC) and eyes open (EO) conditions were acquired using a BrainAmp DC amplifier (Brain Products, Munich, Germany) with a 64-channel EEG system. Participants were asked to sit in a comfortable chair and to remain relaxed during the EEG recording. A conductive gel was used to keep the electrode impedances below 5 kΩ. During the recording, participants were instructed to look at a fixation throughout the process. Using a sampling rate of 5,000 Hz, the EEG signals were referenced online to FCz. The EEG preprocessing was conducted offline with MATLAB (R2018a, The Mathworks Inc., Natick, MA, United States) using a home-made script that was structured based on that of EEGLAB (Delorme and Makeig, 2004) with the following steps: (1) down-sampling to 250 Hz; (2) removal of representative artifacts by visual inspection; (3) application of a zero-phase finite impulse response filter for band-pass filtering between 1 and 45 Hz and removal of 50 Hz line noise and harmonics by notch filtering; (4) rejection of corrupted channels and spherical interpolation of rejected channels; and (5) application of independent component analysis to remove remaining artifacts, including blinks, electrocardiogram signals, and high-frequency sustained muscle artifacts.

### EEG Source Localization and Connectivity Analyses

EEG source localization and source spatial connectivity analyses were carried out using customized scripts and public toolboxes (including EEGLAB and Brainstorm) in MATLAB (Tadel et al., 2011). Specifically, the head model of the three-layer boundary element was calculated, and a rotating dipole with 3,003 vertices was then produced on the cortical surface. The lead field matrix of the dipole activities related to the EEG was acquired, and the minimum norm estimation (MNE) was used in the inverse model; the dipole direction is limited to the normal of the cerebral cortex (Lin et al., 2006). Functional connectivity based on the dWPLI correlation was computed among 31 regions of interest (ROIs) in the Montreal Neurological Institute space, stemmed from independent components analysis parcellation of functional magnetic resonance imaging connectivity of 38 healthy individuals used in a previous study (Toll et al., 2020). The dWPLI can be defined as:

(1) $${\hat{\Omega }}^{w}=\frac{\sum_{j=1}^{N}\sum_{k=j+1}W_{j,k}d\left(X_{j},X_{k}\right)}{N\left(N-1\right)\hat{W}}$$

where $\hat{W}$ represents the average weight, i.e., the weight normalization expressed as $\frac{1}{N\,\left(N-1\right)}\,\sum_{j=1}^{N}\sum_{k=j\,1}W_{j,k}$. The dWPLI ranges from 0 to 1. The higher the dWPLI value, the higher the coupling between neural oscillations (Vinck et al., 2011). Finally, excluding self-connectivity, 465 unique ROI pairs were calculated in each of the five frequency bands (delta: 1–4 Hz, theta: 5–7 Hz, alpha: 8–12 Hz, beta: 13–30 Hz, gamma: 31–45 Hz).

### Statistical Analyses

Statistical analyses were conducted using the IBM Statistical Package for the Social Sciences (SPSS; IBM Corp., Armonk, NY, United States) Version 25.0 and MATLAB. For analysis of variance, normality was tested by Shapiro–Wilk test; normally distributed data were represented as means and standard deviations, while non-normally distributed data were represented as medians and interquartile ranges. Group differences in age, education level, UPDRS-III score, and PSQI score were compared using independent-samples *t*-tests. Group differences in the disease course, H&Y stage, neuropsychological tests, and LEDD were compared using Mann–Whitney *U* tests, and sex differences between groups were compared using chi-squared tests. Network-based Statistics (NBS) (Zalesky et al., 2010), a nonparametric statistical test controlling for the family wise error rate resulting from multiple comparisons, was used to analyze differences in functional connectivity between the PD-MCI and PD-NC groups based on the following steps: (1) a two-sample *t*-test was conducted at each connection, and generating a test statistic value matrix demonstrating significant functional connectivity differences between the groups; (2) application of a component-forming threshold to obtain a set of supra-threshold matrices, and computing the size of the connected component in the matrices; (3) generating an empirical null distribution of largest component sizes by mean of randomly permuting the PD-MCI and PD-NC group membership, calculating the test statistic of interest, storing the largest component sizes, and repeating for 5,000 times; (4) estimating the *p-*value of the connected element (i.e., the proportion of the empirical null component sizes that was greater than the actual value), and *p <* 0.05 was defined as a significant difference between the groups. Partial correlation analysis was performed to evaluate the correlations between the seed-based functional connectivity and the MoCA score (as well as the MoCA subdomains, including executive, memory, visuospatial, language, and attention functions), adjusting for sex, age, and education level. To measure the power of the seed-based functional connectivity differences, receiver operating characteristic (ROC) curve analysis was performed. The statistical significance level was set at *p* < 0.05.

## Results

### Demographic and Clinical Characteristics

Based on the above inclusion and exclusion criteria, 71 patients were initially included in this study; however, three participants were removed due to poor signal quality. Consequently, the final sample included 68 patients (37 males and 31 females), with 32 patients belonging to the PM-MCI group and the other 36 individuals to the PD-NC group. Relative to the PD-NC group, the PD-MCI group presented significantly lower MoCA scores (as well as the MoCA subdomains, including executive, memory, visuospatial, language, and attention functions; see Supplementary Table 1) and education levels, and a significantly higher mean age. No other significant differences were found between the two groups. The detailed demographic and clinical characteristics, as well as the statistical results, are shown in Table 1.

**TABLE 1:** Main demographic and clinical characteristics in each group.

	All	PD-MCI	PD-NC	t/z/χ^2^	*P* value
N	68	32	36		
Sex (M/F)	37/31	17/15	20/16	0.040	0.841
Age (year)	63.97 ± 9.09	66.67 ± 7.33	61.50 ± 9.92	–2.443	0.017*
Education (year)	8.59 ± 4.32	6.27 ± 4.06	10.72 ± 3.39	4.961	<0.001*
Duration (year)	2.5 (1.5, 5)	3 (1.5, 5.375)	2 (1, 5)	–0.321	0.748
UPDRS-III score	23.16 ± 11.30	24.79 ± 11.49	21.67 ± 11.08	–1.149	0.255
H&Y stage	2 (1.5, 2.5)	2 (1.5, 2.5)	1.75 (1, 2.375)	–1.053	0.292
PSQI score	9.36 ± 4.28	9.65 ± 4.33	9.05 ± 4.31	–0.431	0.669
LEDD (mg)	258.335 (118.75, 400)	233.335 (0,346.875)	275 (176.25, 446.875)	–1.044	0.297
HAMA	9 (6, 14.5)	9 (6, 17)	9 (5.25, 12.75)	–0.801	0.423
HAMD	11 (6.5, 15)	11 (4.5, 16)	11 (7, 14.75)	–0.096	0.923
MoCA	24 (19, 27)	19 (13.5, 22)	26.5 (25, 27.75)	–7.159	<0.001*

*p < 0.05 (comparison between PD-MCI and PD-NC group). HAMA, Hamilton Anxiety Rating Scale; HAMD, Hamilton Depression Rating Scale; H&Y, Hoehn and Yahr; LEDD, levodopa equivalent dose daily; MoCA, Montreal Cognitive Assessment; PD-MCI, Parkinson’s Disease with mild cognitive impairment; PD-NC, Parkinson’s Disease with normal cognition; PSQI, Pittsburgh Sleep Quality Index; UPDRS-III, Unified Parkinson’s Disease Rating Scale-III.

### Seed-Based Functional Connectivity With Bilateral PMFG

**FIGURE 1:**
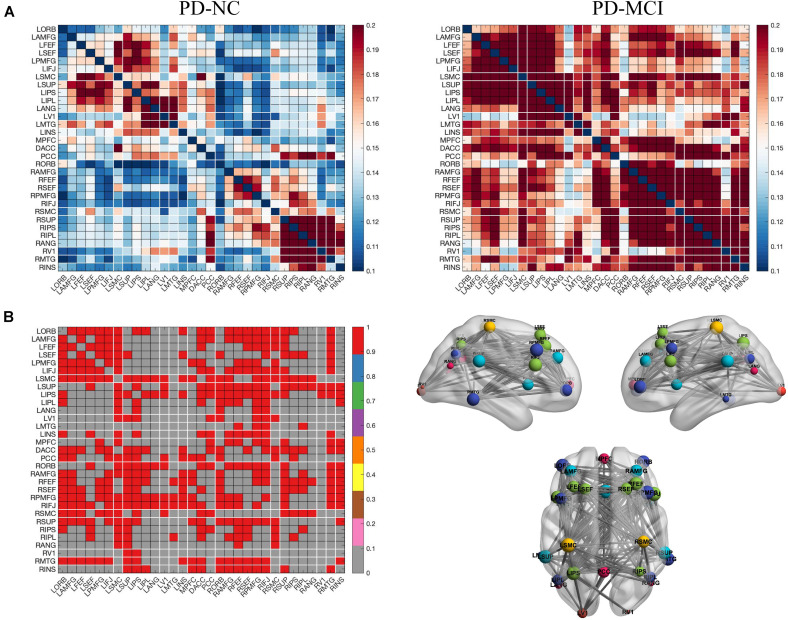
Left and right PMFG-based functional connectivity changes in the theta frequency band under EC condition in PD-related mild cognitive impairment. **(A)** Functional connectivity matrices of the PD-MCI and PD-NC groups. **(B)** NBS analysis of group differences (PD-MCI group > PD-NC group, including the left PMFG and the right PMFG-based functional connectivity). Differential functional connectivity between the two groups (left), and 3D brain connectivity patterns showing the left side, right side, and the upper view, respectively (right). In **(B)**, the red matrix element represents the variant edges of the NBS analysis and the size of the node indicates the sum of the number of surviving edges for each node. EC, eyes closed; PD-MCI, Parkinson’s disease with mild cognitive impairment; PD-NC, Parkinson’s disease with normal cognition; NBS, network-based statistics; L, left; R, right; ORB, orbitofrontal cortex; AMFG, anterior division of the middle frontal gyrus; FEF, frontal eye field; SEF, supplementary eye field; PMFG, posterior division of the middle frontal gyrus; IFJ, inferior frontal junction; SMC, sensorimotor cortex; SUP, supramarginalgyrus; IPS, intraparietal sulcus; IPL, inferior parietal lobule; ANG, angular gyrus; MTG, middle temporal gyrus; V1, primary visual cortex; INS, insular cortex; MPFC, medial prefrontal cortex; DACC, dorsal anterior cingulate cortex; PCC, posterior cingulate cortex.

**FIGURE 2:**
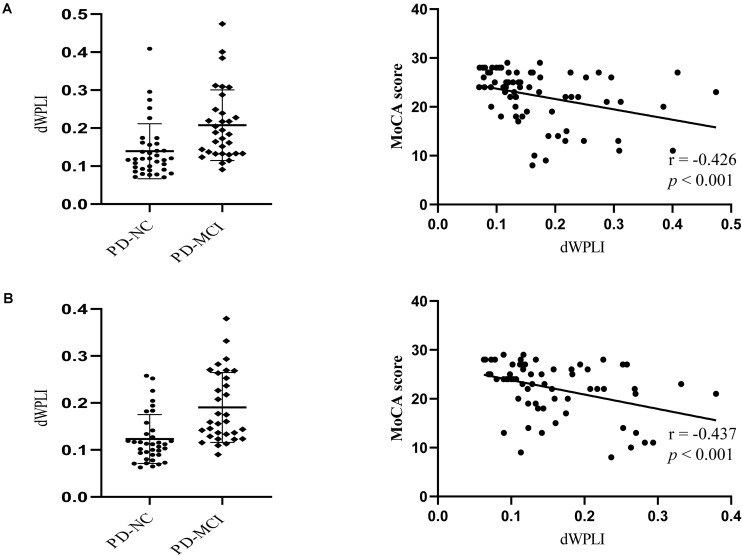
The MoCA scores are negatively correlated with the left PMFG-based functional connectivity change **(A)** and the right PMFG-based functional connectivity change **(B)** after adjusting for sex, age, and education level. Scatterplot depicting seed-based functional connectivity significantly differed between the groups. dWPLI, debiased weighted phase lag index; MoCA, Montreal Cognitive Assessment; PD-MCI, Parkinson’s disease with mild cognitive impairment; PD-NC, Parkinson’s disease with normal cognition.

NBS analysis revealed stronger brain-wide functional connectivity in the PD-MCI group than in the PD-NC group in the theta band under EC condition but not in any other bands or conditions. Specifically, components, including 216 edges connected to 31 nodes, showed enhanced connectivity strength between the groups, involving the visual, somatosensory, dorsal attention, default mode (DMN), frontoparietal control, and ventral attention networks (Figures 1A,B). When the left and right PMFG were selected as seed ROIs, the identified clusters involved multiple networks; for the left PMFG, the identified clusters involved the somatosensory, dorsal attention, default mode, frontoparietal control, and ventral attention networks; while the identified clusters for the right PMFG involved the visual, somatosensory, dorsal attention, default mode, frontoparietal control, and ventral attention networks (Figure 1B). For all patients with PD, we next calculated the nodal functional connectivity for the selected ROIs as the average functional connectivity between the bilateral PMFG and all other identified clusters revealed by NBS. The seed-based functional connectivity significantly increased in the PD-MCI group, and the MoCA scores were negatively correlated with the left and right PMFG-based functional connectivity values (*r* = −0.426, *p* < 0.001 and *r* = −0.437, *p* < 0.001, respectively), after adjusting for sex, age, and education level (Figure 2). Concerning the subdomains of the MoCA, poorer visuospatial function was associated with higher right PMFG-based functional connectivity (*r* = −0.335, *p* = 0.005) and poorer attention function was associated with higher left and right PMFG-based functional connectivity (*r* = −0.380, *p* = 0.001 and *r* = −0.256, *p* = 0.035, respectively) after adjusting for sex, age, and education level (Supplementary Figure 1).

### Prediction of Cognitive Outcome

**FIGURE 3:**
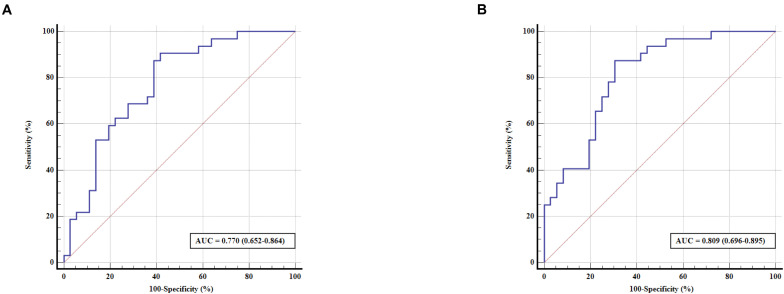
ROC curve for predicting cognitive status in patients with PD based on the significant functional connectivity with the left PMFG **(A)** and right PMFG **(B)**. The x-axis indicates the percentage of (100 – specificity), and the y-axis indicates the percentage of sensitivity. AUC, area under the receiver operating characteristic curve; ROC, receiver operating characteristic; PMFG, posterior division of the middle frontal gyrus.

Enter logistic regression analysis was performed for mild cognitive impairment based on the significant functional connectivity with the left and right PMFG, respectively, adjusting for sex, age, and education level. The logistic regression analysis results are shown in Table 2. The significant functional connectivity with the left and right PMFG were both independent risk factors for mild cognitive impairment in patients with PD [odds ratio (OR): 2.949, 95% confidence interval (C) 1.294–6.725, *p* = 0.01; OR: 11.278, 95% CI: 2.578–49.335, *p* = 0.001, respectively, per 0.1U]. In contrast to these findings, the logistic regression analysis based on the significant power spectral density from univariate analysis in the PMFG region failed to detect any significant results after adjusting for sex, age, and education level (data not shown). The ROC analysis results are shown in Figure 3 and Table 3. The area under the ROC curve (AUC) of significant functional connectivity with the left PMFG was 0.770, with a sensitivity of 87.50% and a specificity of 61.10%. The AUC of significant functional connectivity with the right PMFG was 0.809, with a sensitivity of 87.50% and a specificity of 69.40%.

**TABLE 2:** Logistic regression analysis for cognitive status in PD patients based on the significant functional connectivity with the left PMFG and right PMFG, respectively.

	B	S.E.	Wald	*p* value	OR	95% CI
Left PMFG, per 0.1 U	1.082	0.421	6.616	0.01	2.949	1.294–6.725
Right PMFGI, per 0.1 U	2.423	0.753	10.354	0.001	11.278	2.578–49.335

*CI, confidence interval; OR, odds ratio; PMFG, posterior division of the middle frontal gyrus.*

**TABLE 3:** Receiver operating characteristic results predicting cognitive status in PD patients based on the significant functional connectivity with the left PMFG and right PMFG, respectively.

Variable	AUC	95% CI	Sensitivity (%)	Sensitivity (%)
Left PMFG	0.770	0.652–0.864	87.50	61.10
Right PMFG	0.809	0.696–0.895	87.50	69.40

*AUC, area under the receiver operating characteristic curve; CI, confidence interval; PMFG, posterior division of the middle frontal gyrus.*

## Discussion

Using resting-state EEG dWPLI measurement, the present study revealed abnormal PMFG-based functional connectivity associated with cognitive impairment in patients with PD in the theta frequency bands under the EC condition. Logistic regression and ROC curves analyses found that such abnormal functional connectivity was an independent risk factor for cognitive impairment and could be used to discriminate the cognitive condition of patients with PD. Critically, based on PMFG regional power spectral density, a measure commonly used in previous EEG studies (Mostile et al., 2019), the logistic regression analysis did not identify any risk factors.

### Cognitive Disruption in PD and Changes in Brain Connectivity

Cognitive dysfunction is pervasive in PD and negatively impacts patients’ quality of life (Hanagasi et al., 2017). Therefore, investigations of the clinical features or predictive biomarkers for this condition in an early stage are essential. Electroencephalography functional connectivity could make a meaningful contribution to this procedure, given that altered signal rhythms in PD-MCI patients are related to underlying neuropathological changes (Geraedts et al., 2018). Several studies have reported alterations in functional connectivity in patients with PD compared with healthy controls, and these abnormalities are exacerbated by cognitive impairments (Bertrand et al., 2016; Sanchez-Dinorin et al., 2021). The PD-MCI patients in the current study had stronger brain-wide functional connectivity in the theta frequency band compared with PD-NC patients. Different EEG bands have different normal functions and anatomical connections. Correspondingly, increased slow frequency (e.g., in the theta and delta bands) activity is characteristic of dysfunction in the diffuse gray matter in the cortical and subcortical regions along with local deafferentation in the cerebral cortex (Steriade et al., 1990). Thus, the hyper-synchronization in this frequency band is probably the result of a compensatory mechanism through which additional brain regions are enlisted to maintain cognitive performance (Gorges et al., 2015).

The DLPFC, thought to be a part of the limbic cortical-striatal-pallidal-thalamic network, has been proven to be one of the primary regions of interest for neuromodulation, and various imaging studies have demonstrated direct or indirect relationships between cognitive function and the DLPFC (Gratwicke et al., 2015; Rosero Pahi et al., 2020). In terms of the nodes located in the DLPFC, the bilateral PMFG are widely used as accessible cortical stimulation sites for NIBS (Chen et al., 2013). What happens to the brain connectivity patterns that interact with these sites? Our results demonstrate that the identified clusters involve multiple networks, implying that cognitive functions require coordinated communication among multiple brain regions. In support of these findings, a systematic review of neuroimaging studies investigating non-motor symptoms in PD suggested that several anatomically separated brain areas, including the sensorimotor and executive networks (particularly, the DLPFC and the anterior cingulate cortex), as well as the DMN, visual, auditory, salience (insular), frontal, parietal, and temporal networks, are all functionally connected during resting states, and the functional connectivity between these brain regions plays a pivotal role in coordinating complex cognitive processes (Prell, 2018). Furthermore, abnormal functional connectivity was associated with the MoCA score, which represents a global test scale for cognitive function, indicating that abnormal PMFG-based functional connectivity is involved in cognitive performance in patients with PD. Notably, Nissim et al. found that paired active-transcranial direct current stimulation (tDCS) over the left DLPFC together with cognitive training significantly increased working memory performance, as well as functional connectivity between the left DLPFC and right inferior parietal lobule in older adults (Nissim et al., 2019), which may also indicate that the DLPFC connectivity pattern plays a crucial role in cognitive function. On the other hand, our results also corroborate previous studies of changed functional connectivity in patients with PD that is related to their cognitive impairments (Bertrand et al., 2016; Hassan et al., 2017; Chaturvedi et al., 2019; Sanchez-Dinorin et al., 2021). Among them, Hassan et al. demonstrated that lower edge-wise connectivity in the alpha1 and alpha2 frequencies was associated with lower cognitive state, particularly in the frontotemporal areas (Hassan et al., 2017). While another study using the phase-locking value found enhanced functional connectivity within the frontal regions over all frequency bands, and changes in the delta and theta frequency bands were associated with poorer cognitive performance in PD patients (Sanchez-Dinorin et al., 2021). Indicators of functional connectivity determined by different algorithms may disclose different underlying neural mechanisms. The results of functional connectivity measures depend on the type of functional connectivity measures and the cognitive behavioural measurement instrument used (Geraedts et al., 2018). Notably, the dWPLI correlation used in this study, could potentially exclude significant zero-lag connectivity caused by a mixture of true and spurious correlations; additionally, it is expected to detect real-time lagged functional couplings of brain source activities (Lau et al., 2012).

Furthermore, patterns of altered functional connectivity may be domain-specific, and relevant studies that consider functional connectivity in the context of cognitive domains are rare. While we only used the MoCA score as a measure of global cognitive function in the current study, we attempted to correlate the subitem scores of the MoCA with the significant functional connectivity in order to preliminarily explore the relationship between specific cognitive domain functions and the changes of PMFG-based functional connectivity. The performances of visuospatial and attention functions are associated with PMFG-based functional connectivity. These findings confirm the discoveries of a recent study: increased frontal synchronization of slow oscillations predicts global cognitive and visuospatial functions (Sanchez-Dinorin et al., 2021). In addition, another study found that attention and memory correlated significantly with the phase lag index in the beta and theta frequency bands, respectively (Chaturvedi et al., 2019). Nevertheless, as we did not have extensive reliable assessment measurements for specific cognitive domains, conclusions about specific cognitive domains need to be interpreted with caution and warrant further research.

### EEG Measures Predict Cognitive Impairment in PD Patients

The reduction in EEG background frequency has been found to be associated with phosphorylated α-synuclein in autopsy tissue of the posterior cingulate cortex (Caviness et al., 2016), and several studies have also suggested that EEG features (frequency and power features of special regions) could be used to distinguish the severity of cognitive dysfunction or longitudinal cognitive decline in patients with PD using various approaches, including machine learning, which confirms the reliability and reproducibility of EEG in identifying cognitive impairment (Arnaldi et al., 2017; Betrouni et al., 2019; Zhang et al., 2021). Together, these findings provide strong evidence that EEG measures can be used as reliable biomarkers of cognitive decline in patients with PD. However, these studies only used the frequency and power features of EEG rather than functional connectivity that may better reflect the complexity of information processing in the cerebral cortex and may therefore be more closely related to behavior (Bullmore and Sporns, 2012). As described above, brain connectivity would be expected to disclose the properties of brain processing, and current studies have begun to address the issue of changes in brain connectivity, which broadens our understanding of the neural mechanisms of neurodegenerative diseases. Our results reveal that significant functional connectivity with the bilateral PMFG are independent risk factors for mild cognitive impairment in patients with PD, and, in addition, the ROC curve analysis demonstrated that the observed abnormalities of PMFG functional connectivity showed robust discriminative power when determining the cognitive condition in PD. Thus, the presence of such changes could be used as potential markers for identifying cognitive impairment in patients with PD. This corresponds to the higher theta band phase lag index in PD-MCI patients reported by Chaturvedi et al. (Chaturvedi et al., 2019). However, we paid more attention to the brain connectivity related to NIBS targets, which is essential for exploring the neural mechanisms and therapeutic mechanisms of diseases.

### Clinical Significance

Generally, neuropsychological assessments are used to define the status and severity of cognitive impairment in patients with PD. However, it must be acknowledged that neuropsychological assessments require specialized neuropsychological experts, which is both time-consuming and requires good patient cooperation. Resting-state EEG is easily accessible with high test-retest reliability and objectively reflects the pathophysiological functioning of the brain (Salinsky et al., 1991). Furthermore, this procedure can be repeated multiple times without learning bias. Using this procedure, our study found that PMFG-based connectivity could predict the cognitive condition of patients with PD. Regarding clinical considerations, these findings hold promise as practical adjuncts to neuropsychological studies. Moreover, it may be possible to improve cognitive function in patients with PD by adjusting the EEG functional connectivity in specific regions.

This study has several limitations. First, it should be noted that we used the MoCA score as a global cognitive function measure to determine the level of cognition and cognitive impairment that meets the level I diagnostic criteria of PD-MCI; this measure does not assess specific cognitive domains and may, therefore, provide less diagnostic accuracy. To mitigate this issue, the diagnosis of idiopathic PD, as well as the determination of cognitive status in all included patients, were unanimously decided by movement disorder specialists and neuropsychological experts. Second, the alterations in connectivity observed in the present study are outwardly not specific to PD-MCI, as an increased slowing of activities is also common to other types of dementia. However, the pathophysiological mechanisms of dementias with different etiologies can be reflected through types of different brain connectivity; this aspect requires more extensive research in the future. Third, because of the heterogeneity of PD, it is difficult for a single marker to achieve an accurate diagnosis, and the combination of multimodal markers may improve the accuracy of diagnosis. Consequently, it would be of great significance to further explore multimodal markers for cognitive impairment associated with PD. Fourth, the current study is cross-sectional, and future longitudinal studies are warranted. It is expected that the methodology established in this study can be extended by integrating follow-up data to further predict the progression of cognitive impairment in patients with PD. In addition, the current study lacks an age-matched healthy control group, which would be important to highlight unique features in the PD-NC group. Although our purpose was to identify patients with cognitive impairment from PD patients, future in-depth studies incorporating data from the control group are warranted.

## Conclusion

We found abnormal PMFG-based functional connectivity patterns associated with cognitive impairment in patients with PD exclusively in the theta frequency bands under EC condition and demonstrated that this abnormal functional connectivity was an independent risk factor for cognitive impairment in PD. As such, abnormal PMFG-based functional connectivity patterns have the potential to act as reliable biomarkers for identifying cognitive impairment in patients with PD. These results provide a direction for elucidating the neuropathology of PD-MCI and the mechanism of NIBS.

## Data Availability Statement

The raw data supporting the conclusions of this article will be made available by the authors, without undue reservation.

## Ethics Statement

The studies involving human participants were reviewed and approved by the Institutional Research Ethics Committee of Shenzhen People’s Hospital. The patients/participants provided their written informed consent to participate in this study.

## Author Contributions

YG, MC, and GD: conception and study design. XlS, LZ, XS, SC, and XyL: data collection or acquisition. MC, GD, and XlS: data analysis and results interpretation. MC and GD: drafting the manuscript. LZ, XgL, and YG: revising the manuscript. All authors read and approved the final manuscript.

## Conflict of Interest

The authors declare that the research was conducted in the absence of any commercial or financial relationships that could be construed as a potential conflict of interest.
