# Supplementary material for: Identifying Mild Cognitive Impairment in Parkinson’s Disease With Electroencephalogram Functional Connectivity
Source: Front Aging Neurosci. 2021 Jul 1;13:701499. doi: 10.3389/fnagi.2021.701499 (PMC8281812; doi:10.3389/fnagi.2021.701499)
Supplement: Supplementary file 1 [file Data_Sheet_1.docx]

Supplementary Material

# Supplementary Figures and Tables

## Supplementary Tables

Supplementary Table 1. MoCA subdomain scores in each group.

|  | All | PD-MCI | PD-NC | z | *P* value |
| --- | --- | --- | --- | --- | --- |
| N | 68 | 32 | 36 |  |  |
| Executive | 1 (0,1) | 0 (0,1) | 1 (0,1) | -2.640 | 0.008^*^ |
| Memory | 3 (1,4) | 1 (0,3) | 3 (3,4) | -4.570 | <0.001^*^ |
| Visuospatial | 3 (2,4) | 2 (0,2.75) | 4 (3,4) | -5.962 | <0.001^*^ |
| Language | 2 (1,3) | 1 (1,2) | 2.5 (2,3) | -4.340 | <0.001^*^ |
| Attention | 6 (5,6) | 5 (3,6) | 6 (6,6) | -4.205 | <0.001^*^ |

## ^*^*p*<0.05(comparison between PD-MCI and PD-NC group). MoCA, Montreal Cognitive Assessment; PD-MCI, Parkinson’s Disease with mild cognitive impairment; PD-NC, Parkinson's Disease with normal cognition.

## Supplementary Figures


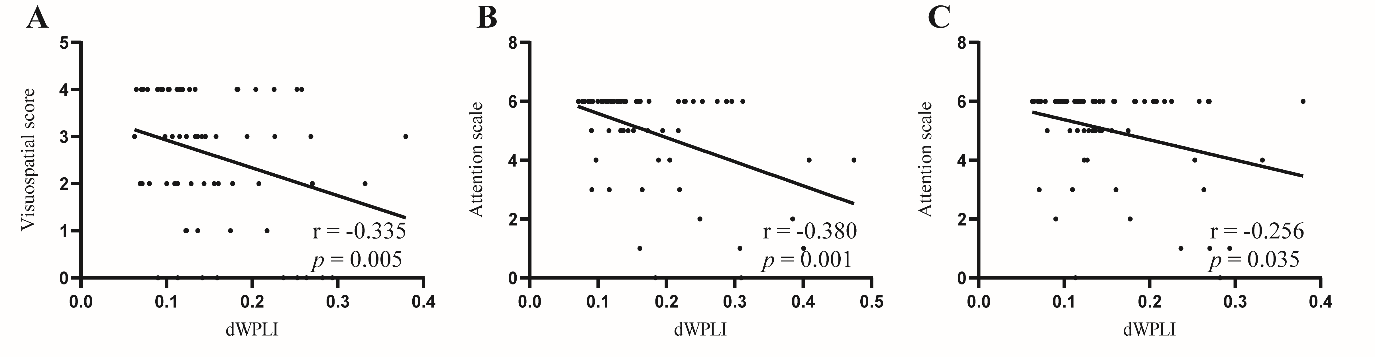


**Supplementary Figure 1.** The visuospatial function scores are negatively correlated with the right PMFG-based functional connectivity change (A), and the attention function scores are negatively correlated with the left (B) and right (C) PMFG-based functional connectivity changes, adjusting for sex, age, and education level.

dWPLI, debiased weighted phase lag index; PMFG, posterior division of the middle frontal gyrus.
